# DAILY SLEEP HEALTH AMONG WORKING INFORMAL CAREGIVERS OF OLDER ADULTS: DELVING INTO CAREGIVING DEMANDS

**DOI:** 10.1093/geroni/igae098.2740

**Published:** 2024-12-31

**Authors:** Eunae Cho, Tuo Yu Chen

**Affiliations:** National Chengchi University, Taipei, Taipei, Taiwan (Republic of China); Taipei Medical University, Taipei, Taipei, Taiwan (Republic of China)

## Abstract

Caregiving demands are a key stressor, but little is known about the relationship between caregiving demands and sleep health among working informal caregivers of older adults. We conducted a 5-day daily diary study to examine the relationship between caregiving demands and sleep health among working informal caregivers of older adults in Singapore (N=193). For each day, we examined ‘anticipated caregiving difficulty’ in the morning; in the evening, we assessed ‘total hours spent on caregiving,’ ‘total number of care activities (e.g., personal care, toileting, running errands),’ and ‘experienced caregiving difficulty.’ We further explored their interaction effects on sleep health. Covariates included sociodemographic status, self-rated health, depressive symptoms, and care recipients’ functional status. We found that anticipating more caregiving difficulty in the morning was significantly associated with poor sleep health in the evening on top of covariates and actual caregiving demands. We also found a significant interaction between ‘anticipated caregiving difficulty’ and ‘experienced caregiving difficulty.’ Specifically, the relationship between experienced caregiving difficulty in the evening and poorer sleep health was stronger among individuals who anticipated a lower level of difficulty that morning (1SD-lower). Individuals who anticipated a higher level of caregiving difficulty in the morning (1SD-higher) had worse sleep health in the evening regardless of the level of caregiving difficulty experienced during the day. Our findings suggest that the impacts of ‘anticipated caregiving difficulty’ on sleep health go beyond actual caregiving difficulty experienced. The mismatch between anticipated and experienced caregiving difficulty plays a role in sleep health at night.

